# Ophthalmic Manifestations of Acute Leukemia

**DOI:** 10.7759/cureus.3837

**Published:** 2019-01-07

**Authors:** Mohammad Uzair Hafeez, Muhammad Hassaan Ali, Nimra Najib, Muhammad Hammad Ayub, Kaleem Shafi, Mubashar Munir, Nadeem Hafeez Butt

**Affiliations:** 1 Department of Ophthalmology, Jinnah Hospital Lahore (JHL)/Allama Iqbal Medical College (AIMC), Lahore, PAK; 2 Department of Ophthalmology, University of California at Los Angeles/ Stein Eye Institute, Los Angeles, USA

**Keywords:** ophthalmic manifestations, leukemia, acute lymphocytic leukemia, acute myelogenous leukemia, frequency

## Abstract

Introduction

Ocular involvement in leukemia may occur because of direct leukemic infiltration or because of secondary ophthalmic involvement as a result of abnormalities related to blood like anemia, thrombocytopenia, and leukocytosis. In some patients with leukemia, ophthalmic signs can precede the systemic features and can help in early diagnosis of systemic leukemia. Due to the scarcity of data on this topic from Pakistan, we conducted this study to determine the pattern of ocular involvement in patients with leukemia presenting in our settings.

Methods

This cross-sectional study was conducted in a tertiary care hospital of Pakistan over a period of one year. The study comprised of both newly diagnosed and follow-up patients of acute leukemia of age more than 15 years. Patients underwent detailed ophthalmic anterior and dilated posterior segment examination. Patient’s demographic profile, type of leukemia, chemotherapy status, and hematologic findings were also documented.

Results

There were 97 leukemic patients in the study with 55 (56.7%) males and 42 (43.3%) females. Various ophthalmic manifestations were observed in 47 (48.45%) patients. Forty-two (43.3%) were diagnosed cases with acute lymphocytic leukemia (ALL) and 55 (56.7%) suffered from acute myelogenous leukemia (AML). Ophthalmic manifestations were present in 29 patients of AML (52.7%) and 18 patients of ALL (42.85%). Ocular involvement was significantly more common in newly diagnosed (n=32) as compared with follow-up patients (n=15) (p-value = 0.032). Posterior segment (n=48) was the most common site of ocular involvement (n=48, 49.5%) with retinal hemorrhages seen in 40 patients (41.2%) and papilloedema in seven cases (7.2%). Thirty-three (70.2%) out of 47 patients with ophthalmic manifestations were asymptomatic while 14 (29.8%) had ocular symptoms at the time of initial presentation.

Conclusion

Ophthalmic manifestations were present in about half of the patients with leukemia. Ocular involvement was more prevalent in newly diagnosed cases and in patients with AML.

## Introduction

Leukemia is a malignant neoplasm of the bone marrow which results in abnormal production of white blood cells and affects multiple organs of the body. It is one of the most common neoplasms [1]. Leukemia can be acute or chronic. Acute leukemias are further categorized as acute myelogenous leukemia (AML) and acute lymphocytic leukemia (ALL) [2]. Anemia, infection, hemorrhage, and organ infiltration are the common features of acute leukemias [3].

Ocular involvement in leukemia may occur due to direct leukemic infiltration or because of secondary ophthalmic involvement as a result of abnormalities related to blood like anemia, thrombocytopenia, and leukocytosis or because of chemotherapy or immunosuppression [4]. Direct leukemic infiltration can involve orbit, anterior segment, uvea, and the optic nerve. It can cause proptosis, cranial nerve palsies or papilledema. Common indirect ophthalmic manifestations are retinal, pre-retinal or vitreous hemorrhages, infections, and retinal venous occlusions [5]. In some patients with leukemia, ophthalmic signs can precede the systemic features and can help in early diagnosis of systemic leukemia [6]. Awareness of ophthalmic signs of leukemia has significant importance as the eye is the only structure where the involvement of nerves and blood vessels affected by leukemia can be directly seen [7].

Leukemia-related ophthalmic manifestations can adversely affect vision and have an important role in indicating the survival prognosis of systemic leukemia [8]. Therefore, a detailed ophthalmic evaluation should be considered in all patients with acute leukemia [9-10]. Due to the scarcity of data on this topic from our country, we conducted this study to determine the pattern of ocular involvement in patients with leukemia.

## Materials and methods

This cross-sectional study was conducted in the Department of Ophthalmology, Jinnah Hospital, Lahore, Pakistan, a tertiary care multi-disciplinary hospital affiliated with Allama Iqbal Medical College, University of Health Sciences, Lahore, Pakistan. The study was approved by the Ethical Review Board of the Allama Iqbal Medical College and was conducted following the ethical principles laid down in the Declaration of Helsinki. The study was conducted from February 2017 to January 2018 and included patients with acute leukemia of age greater than 15 years, admitted in the Oncology department of Jinnah Hospital, Lahore. Both newly diagnosed and follow-up patients were included in this study. Informed written consent was obtained from all the patients. Patients with opacities in the ocular media and with a history of diabetes mellitus, acquired immunodeficiency syndrome (AIDS), sickle cell disease and retinal vascular disease were excluded from the study. Leukemia was diagnosed by consultant hematologist on the basis of bone marrow biopsy. Immunophenotyping by flow cytometry was performed on all bone marrow samples to differentiate AML from ALL.

The ophthalmic examination consisted of a Snellen chart for best corrected visual acuity and anterior segment evaluation by slit lamp biomicroscope. Direct and indirect ophthalmoscopes and slit lamp biomicroscopes with fundus lens were used for detailed dilated fundus examination. Bedridden patients were evaluated on the bedside using direct and portable indirect ophthalmoscopes.

The data was analyzed using Statistical Package for Social Sciences (SPSS) version 20.0 (IBM, Chicago, IL, USA). Descriptive variables were calculated as frequencies and percentages whereas quantitative variables were analyzed as mean (SD). Chi-square test was used to analyze the effect of qualitative variables whereas student's t-test was used to see the effect of quantitative variables on leukemic findings with p-value <0.05 considered as statistically significant.

## Results

There were 97 patients in the study with 55 (56.7%) males and 42 (43.3%) females with a female to male ratio of 1:1.31. Forty-two patients (43.3%) were diagnosed with ALL and 55 (56.7%) patients suffered from AML. Forty-seven out of 97 patients (48.5%) had ophthalmic manifestations at the time of presentation. Out of these, 27 (49.1%) were males and 20 (47.6%) were females (p=0.886) (Table 1).

**Table 1:** Prevalence of ocular manifestations in acute myelogenous leukemia and acute lymphoblastic leukemia according to gender

Leukemia	Gender	Ophthalmic Manifestation		Total	p-value
		Present	Absent		
AML	Female	12	12	24	Chi-sqaure value = 0.127, P = 0.721
	Male	17	14	31	
	Total	29	26	55	
ALL	Female	8	10	18	Chi-sqaure value = 0.032, P = 0.857
	Male	10	14	24	
	Total	18	24	42	
Total	Female	20	22	42	Chi-sqaure value = 0.021, P = 0.886
	Male	27	28	55	
	Total	47	50	97	
AML: Acute myelogenous leukemia; ALL: Acute lymphocytic leukemia					

Thirty-two (68.1%) patients were newly diagnosed and 15 (42.3%) were follow-up patients (p=0.045). Ophthalmic manifestations were more common in patients with AML (52.7%) as compared to ALL (42.85%) and in newly diagnosed (57.1%) as compared to follow-up patients (36.6%) (Table 2).

**Table 2:** Ophthalmic manifestations in newly diagnosed & follow-up patients

Admission Type	Ophthalmic Manifestations		Total	p-value
	Present (n, %)	Absent (n, %)		
New	32, 57.1	24, 42.9	56	Chi-sqaure value = 4.005 p = 0.045
Follow-Up	15, 36.6	26, 63.4	41	
Total	47, 48.5	50, 51.5	97	

A total of 54 ophthalmic manifestations were observed in 47 patients. Some patients had more than one ophthalmic manifestation at the time of presentation in one or both eyes. Posterior segment (n=48) was the most frequent site of ocular involvement. Most common findings in the posterior segment included retinal hemorrhages (41.2%) followed by papilloedema (7.2%). A total of nine patients (16.7%) showed direct leukemic and 45 (83.3%) showed secondary leukemic changes. Thirty (70.2%) patients with ophthalmic manifestations were asymptomatic while 14 (29.8%) had ocular symptoms at the time of presentation (Table 3).

**Table 3:** Various ophthalmic manifestations in acute myelogenous leukemia and acute lymphocytic leukemia

Ophthalmic findings	Leukemia (n = 97)		Total
	AML	ALL	
Intra-retinal Hemorrhages	12	3	15
Retinal Hemorrhages with Roth spots	6	4	10
Pre-retinal Hemorrhages	4	4	8
Papilloedema	4	2	6
Subconjunctival Hemorrhages	2	0	2
No Ophthalmic Manifestations	25	24	49
Intra-retinal and Pre-retinal Hemorrhages	1	0	1
Intra-retinal Hemorrhages and Papilloedema	0	1	1
Intra-retinal and Subconjunctival Hemorrhages	1	2	3
Intra-retinal Hemorrhages and Optic Nerve Infiltration	0	1	1
Intra-retinal Hemorrhages and Cranial Nerve Palsies	0	1	1
Total	55	42	97
AML: Acute myelogenous leukemia ALL: Acute lymphocytic leukemia			

The most commonly observed symptoms were decreased vision (n=12) and red eyes (n=4). Eighty-five patients (87.6%) had good vision ranging from 6/6-6/18 on the Snellen’s chart. Eight patients (8.3%) had impaired vision ranging from 6/24-6/60 whereas four patients (4.1%) had severe loss of vision <6/60 (Table 4).

**Table 4:** Visual acuity of leukemic patients

Vision	Frequency	Percent
Good Vision (6/6-6/18)	85	87.6
Impaired Vision (6/24-6/60)	8	8.2
Loss of Vision (< 6/60)	4	4.1
Total	97	100.0

## Discussion

Leukemia is a malignant bone marrow tumor that affects multiple organs of the body including the eyes. Ocular involvement in leukemia is very common and 9% to 90% prevalence has been reported in various studies [9]. This large difference in the range may be due to the differences in the age groups of study populations or due to the temporary nature of the ophthalmic signs. These signs may disappear after starting the treatment of systemic leukemia or can reappear during the recurrence of the disease [11]. It has been reported that any part of the eye can be affected in leukemic patients [11]. Posterior segment ocular manifestations have been most commonly reported in earlier reports and retinal hemorrhages are the most common posterior segment ocular findings [11].

In the current study, ophthalmic manifestations were present in 48.5% of patients with acute leukemia. The frequency of ocular signs in acute leukemia was similar to the studies of Koshy et al. (51.9%) [10], Khadka et al. (46%) [4], Gawai et al. (52.23%) [5], and Dhasmana et al. (45.1%) [7]. On the other hand, it was significantly less than those reported in the study of Eze et al. (72.7%) [8]. A higher prevalence of ocular manifestations among the Nigerian and African population could be attributed to resource deficient health care settings in Nigeria where patients present late in the course of the disease.

Majority of patients included in our study were males (56.7%). This pattern of male majority in patients with acute leukemia is comparable to that reported in the studies of Bitirgen et al. (60%) [12], Koshy et al. (63.5%) [10], Gawai et al. (61%) [5], and Dhasmana et al. (64.7%) [7]. This trend points to the epidemiological higher prevalence of acute leukemia in the male population. However, we could not see any significant difference in the ocular involvement of males versus females in our study. The studies done in India by Koshy et al. [10] and Gawai et al. showed nearly the same results with regards to gender distribution [5]. Our study revealed significantly more frequent ophthalmic findings in newly diagnosed patients (57.1%) compared to follow-up patients (36.6%) (p=0.032). This could be explained on the basis of reduction in ophthalmic manifestations after starting anti-cancer treatment of systemic leukemia. Ocular signs were more common in patients with AML (AML=52.7%, ALL=42.85%, p>0.05). This was comparable to a study by Gawai et al. (AML=65.5%, ALL=58.8%) [5].

Posterior segment ocular findings (88.9%) were significantly more common than anterior segment manifestations. Most common posterior segment manifestations were hemorrhages, that included pre-retinal hemorrhages, intra-retinal hemorrhages, and retinal hemorrhages with Roth spots. Ophthalmic manifestations were more commonly seen as a consequence of secondary changes in systemic leukemia (83.3%) as compared to the direct ocular involvement in acute leukemia (16.7%). These findings were statistically significant and supported by the studies of Koshy et al. [10], Khadka et al. [4], Gawai et al. [5], and Eze et al. [8].

Eight patients had impaired vision (8.2%) with four patients having papilloedema, two having pre-retinal hemorrhages over the pre-macular area, while two having intra-retinal hemorrhages over macula. A total of four patients had severe loss of vision (4.1%). Of these four patients, one had optic nerve infiltration with intra-retinal hemorrhages, one had papilloedema and two had pre-retinal hemorrhages over the premacular area. Out of the 47 patients with ophthalmic manifestations, 70.2% presented without symptoms while only 29.8% had at least one or more ocular symptoms. Absence of any ocular signs symptoms in patients with acute leukemia has major implications. Studies have shown that ocular involvement in patients with lymphocytic and myelogenous leukemia may be associated with poorer prognosis of systemic leukemia [13]. It can also be a sign of recurrence of the disease and hence may be an indication for the treatment of systemic leukemia [14]. Therefore, it is mandatory to do detailed ophthalmic evaluation in all patients with leukemia [15-21].

## Conclusions

Ocular involvement is seen in almost half of the patients of acute leukemia. Acute leukemia is more common in males and ophthalmic manifestations related to this disease are more prevalent in newly diagnosed patients with AML as compared to ALL. As a majority of the patients with ocular signs have no ocular symptoms, it is recommended to conduct a mandatory ophthalmic examination in all leukemic patients, at the time of diagnosis, to detect ocular complications at an early stage.
